# Genome-resolved insights into arsenic-impacted paddy soil and microcosm-derived microbiomes from West Bengal, India

**DOI:** 10.1128/mra.00059-26

**Published:** 2026-04-27

**Authors:** Shuchishloka Chakraborty, Debarshi Mukherjee, Pinaki Sar

**Affiliations:** 1 Department of Bioscience and Biotechnology, Indian Institute of Technology Kharagpur 30133, Kharagpur, West Bengal, India; Montana State University, Bozeman, Montana, USA

**Keywords:** arsenic, paddy soil microbiome, West Bengal, metagenome-assembled genomes (MAGs), genomic landscape, biogeochemical cycle

## Abstract

This study reports 32 metagenome-assembled genomes (MAGs) reconstructed from arsenic (As)-impacted paddy soils of West Bengal, India, and microcosms from these soil samples. These MAGs, represented by 10 bacterial and 2 archaeal phyla, provided critical insights into the metabolic and biogeochemical potential of microbiomes in a highly As-impacted agroecosystem.

## ANNOUNCEMENT

Soil microbiomes are vital for maintaining soil fertility and productivity (1–4). In As-affected paddy fields of West Bengal, prolonged irrigation with contaminated groundwater is known to affect soil microbial communities (5–8). Here, we present 32 MAGs reconstructed from As-impacted paddy soils and microcosms, providing insights into the microbial community’s genomic and metabolic potential in biogeochemical cycling under As stress.

**Fig 1:**
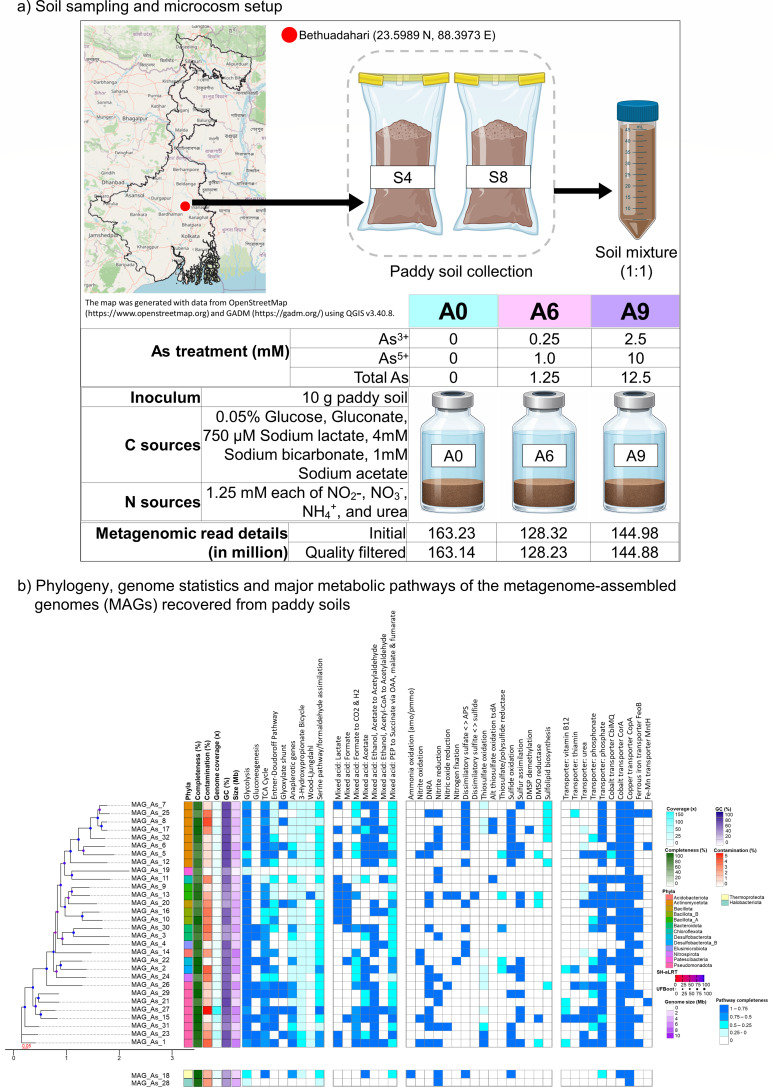
(**a**) Soil sampling and microcosm setup. The map was generated with data from OpenStreetMap (https://www.openstreetmap.org) and GADM (https://gadm.org/) using QGIS v3.40.8, and the soil collection bags, centrifuge tubes, and serum vials were prepared using BioRender (BioRender.com [2025]. BioRender—scientific illustration tool, https://biorender.com, Student trial version). (**b**) Maximum likelihood tree of 30 bacterial and 02 archaeal MAGs recovered from paddy soils. The phylogenetic tree was constructed using a concatenated set of 120 conserved marker genes from each of these MAGs using GTDB-Tk and IQ-TREE3, with branch support values indicated by colored nodes (SH-aLRT bootstrap; scale bar = 0.05 substitutions per site). A heatmap summarizes each MAG’s taxonomic phyla assignment, genome completeness, contamination, genome coverage, GC content, and genome size (Mb), as well as the presence-absence of major pathways involved in biogeochemical cycles, including carbon, nitrogen, sulfur, and the production of short-chain fatty acids. The combined phylogenetic and functional profiles reveal distinct arsenic-responsive microbial lineages, highlighting their potential roles in nutrient cycling in the paddy soil ecosystem.

Arsenic-impacted soils (S4 and S8) previously collected from two paddy fields in Bethuadahari, West Bengal, India (23.5989°N, 88.3973°E) (3, 4) were used in this study. For microcosm setup, these two samples were mixed (1:1, dry weight), and 10 g mixture was added to 40 mL minimal salt medium in 100 mL serum vials (9, 10), supplemented with 1.25 or 12.5 mM total As, or without As. Microcosms were designated as A0 (without As), A6 (1.25 mM As), and A9 (12.5 mM As), and incubated at ~25°C for 60 days (Fig. 1a).

Total DNA from each of three microcosms (0.25 g wet weight, in triplicate) was extracted (DNeasy PowerSoil Pro Kit, QIAGEN). Paired-end libraries were prepared using Illumina DNA Prep (M) Tagmentation Kit and sequenced on Illumina NovaSeq 6000 (2 × 101 bp chemistry) with default parameters. Metagenome sequences of S4 and S8 (3) (BioProject—PRJNA686650), along with three microcosm data sets yielded 108.69 (S4), 114.12 (S8), 163.23 (A0), 128.32 (A6), and 144.98 (A9) million initial reads. These five metagenomic data sets were processed using the metaWRAP pipeline (v1.3.2) (11). Quality control was done using the read_qc module, and host-associated reads were filtered against the GRCh38 (v38) reference genome. Quality-filtered reads were concatenated and co-assembled using MEGAHIT (v1.1.3) (12). Binning was done in universal mode through MetaBAT 2 v2.12.1, MaxBin 2.0 v2.2.6, and CONCOCT v1.0.0 (13–15), followed by bin refinement using the bin_refinement module (11). Genome abundance was estimated as genome copies per million filtered reads using quant_bins. MAGs with >70% completeness and <5% contamination were taxonomically classified with GTDB-Tk (v2.1.1, database r226), and resulting alignments were visualized using IQ-TREE (v3) (16). Functional annotation of the recovered MAGs was performed using eggNOG-mapper (v2.1.12) with eggNOG database (v5.0.2) (17).

Co-assembly of quality-filtered reads from S4 (108.52 million), S8 (113.94 million), A0 (163.14 million), A6 (128.23 million), and A9 (144.88 million) produced 30,0640 contigs (largest: 749,218 bp; N50: 3,184 bp). A total of 32 MAGs, 17 high quality (>90% completeness, <5% contamination) and 15 good quality (>70% completeness, <5% contamination) were recovered. Average completeness of these MAGs was 88.02% (70.39%–99.65%), contamination: 2.13% (0%–4.93%), GC content: 0.58 (0.35–0.73), and N50: 41.48 kb (3.06–194.74 kb) (Table 1). Phylogenetically, they were affiliated with Bacteroidota, Bacillota, Actinomycota, Nitrospirota, Pseudomonadota, Patescibacteria, Acidobacteriota, Halobacteriota, Elusimicrobiota, Thermoproteota, Desulfobacterota, and Chloroflexota (Fig. 1b).

**TABLE 1:** Detailed taxonomy, ENA accession numbers, and statistics of 32 MAGs obtained from As-impacted paddy soils of West Bengal, India

MAG ID	Taxonomy (as per GTDBtk)	ENA accession	Completeness (%)	Contamination (%)	Genome coverage (×)	GC (%)	Size (Mb)	N50 (kb)	No. of contig	Total CDS	RNA
MAG_As_1	Bacteria; Pseudomonadota; Gammaproteobacteria; Burkholderiales; Burkholderiaceae; Cupriavidus; Cupriavidus necator_D	GCA_978015335	88.3	3.765	28.01	66.9	6.37	26	332	5,894	46
MAG_As_2	Bacteria; Desulfobacterota_B; Binatia; UBA9968; UBA9968; DP-20	GCA_978015175	98.68	3.87	27.71	57.8	5.46	48	261	5,192	47
MAG_As_3	Bacteria; Bacteroidota; Bacteroidia; Flavobacteriales; UBA2798; CAMBFX01	GCA_978015145	79.2	2.974	15.18	37.9	2.64	3.1	752	2,251	17
MAG_As_4	Bacteria; Elusimicrobiota; Elusimicrobia; UBA1565; UBA9628	GCA_978015405	91.76	0.374	21.07	64.4	2.62	17	174	2,538	58
MAG_As_5	Bacteria; Actinomycetota; Actinomycetes; Mycobacteriales; Mycobacteriaceae; Rhodococcus; Rhodococcus aetherivorans	GCA_978015245	73.59	0.902	13.14	70.9	4.31	11	493	4,148	44
MAG_As_6	Bacteria; Actinomycetota; Actinomycetota; CALGFH01; CALGFH01; CALGFH01	GCA_978015275	77.48	0.854	28.87	73.4	4.08	4	1,122	4,161	42
MAG_As_7	Bacteria; Actinomycetota; Actinomycetota; UBA4738; HRBIN12	GCA_978015125	88.79	0.854	19.72	70.8	2.42	40	81	2,413	35
MAG_As_8	Bacteria; Actinomycetota; Actinomycetota; UBA4738; HRBIN12; AC-69	GCA_978015165	95.72	4.273	16.24	68	3.53	31	225	3,638	60
MAG_As_9	Bacteria; Bacillota_B; Peptococcia; DRI-13; DRI-13	GCA_978015415	90	0.583	6.89	48.6	2.26	14	192	2,153	46
MAG_As_10	Bacteria; Bacillota_A; Clostridia; Christensenellales; Aristaeellaceae; RUG11247; RUG11247 sp023444095	GCA_978015325	97.98	3.091	13.46	62.1	3.29	20	249	3,010	41
MAG_As_11	Bacteria; Chloroflexota; Anaerolineae; Anaerolineales; Anaerolineaceae; UBA3924	GCA_978015385	74.72	3.03	12.04	60.4	2.82	195	26	2,487	29
MAG_As_12	Bacteria; Actinomycetota; Thermoleophilia; Solirubrobacterales; 70-9	GCA_978015365	84.54	0.985	22.33	69.8	2.52	26	153	2,657	43
MAG_As_13	Bacteria; Bacillota_B; Desulfitobacteriia; Desulfitobacteriales; Desulfitobacteriaceae	GCA_978015305	93.58	2.04	16.47	41	3.32	140	64	3,106	36
MAG_As_14	Bacteria; Acidobacteriota; Blastocatellia; Pyrinomonadales; Pyrinomonadaceae; PSRF01	GCA_978015155	84.25	2.564	22.62	53.9	6.33	13	373	5,543	56
MAG_As_15	Bacteria; Pseudomonadota; Gammaproteobacteria; Enterobacterales; Enterobacteriaceae; Enterobacter; Enterobacter hormaechei_B	GCA_978015105	99.47	0.394	64.44	55.4	4.64	173	42	4,315	74
MAG_As_16	Bacteria; Bacillota_A; Clostridia; Christensenellales; Aristaeellaceae	GCA_978015235	81.37	3.427	4.98	60	4.07	3.5	903	3,812	17
MAG_As_17	Bacteria; Actinomycetota; Actinomycetota; UBA4738; HRBIN12; AC-69	GCA_978015295	97.77	2.849	38.81	67.9	3.19	28	281	3,435	57
MAG_As_18	Archaea; Thermoproteota; Nitrososphaeria; Nitrososphaerales; Nitrososphaeraceae; TA-21	GCA_978015315	98.95	1.941	23.94	35.6	2.16	36	90	2,545	25
MAG_As_19	Bacteria; Patescibacteria; Paceibacteria; UBA9973; UBA9973; UBA9973	GCA_978015345	71.11	0	22.81	42.1	0.63	12	84	634	49
MAG_As_20	Bacteria; Bacillota; Bacilli; Bacillales; Bacillaceae; Metabacillus	GCA_978015215	71.71	2.835	9.00	34.8	4.3	24	282	4,126	28
MAG_As_21	Bacteria; Pseudomonadota; Gammaproteobacteria; REEB76; REEB76	GCA_978015395	79.95	1.427	45.17	72.4	1.97	6.1	394	1,942	36
MAG_As_22	Bacteria; Desulfobacterota; Desulfuromonadia; Geobacterales; Geobacteraceae; Geomonas	GCA_978015265	98.7	0	10.49	61.6	4.28	83	82	3,757	53
MAG_As_23	Bacteria; Pseudomonadota; Gammaproteobacteria; Burkholderiales; SG8-39; RBG-16-66-20	GCA_978015115	94	2.638	69.29	67.4	3.35	30	152	3,464	35
MAG_As_24	Bacteria; Nitrospirota; Nitrospira; Nitrospirales; Nitrospiraceae; Nitrospira_C	GCA_978015375	70.39	3.193	28.99	59	2.1	4.2	340	2,309	24
MAG_As_25	Bacteria; Actinomycetota; Actinomycetota; UBA4738; HRBIN12	GCA_978015225	71.03	3.448	15.00	71.4	2.51	23	146	2,542	41
MAG_As_26	Bacteria; Pseudomonadota; Alphaproteobacteria; Rhizobiales; Rhizobiaceae; Sinorhizobium; Sinorhizobium meliloti_A	GCA_978015285	95.58	1.334	31.92	62.7	5.5	25	362	5,133	50
MAG_As_27	Bacteria; Pseudomonadota; Gammaproteobacteria; Pseudomonadales; Pseudomonadaceae; Pseudomonas_F; Pseudomonas_F sp014700075	GCA_978015205	98	4.931	143.13	64.2	5.9	104	90	5,386	68
MAG_As_28	Archaea; Halobacteriota; Methanosarcinia; Methanosarcinales; Methanosarcinaceae; Methanosarcina; Methanosarcina sp11020u	GCA_978015195	95.06	2.614	9.04	39.2	4.14	13	368	3,508	51
MAG_As_29	Bacteria; Pseudomonadota; Gammaproteobacteria; Xanthomonadales; Xanthomonadaceae; CFH-32150	GCA_978015255	99.65	1.077	15.93	65.5	3.17	120	34	2,933	48
MAG_As_30	Bacteria; Bacteroidota; Ignavibacteria; Ignavibacteriales; SURF-24	GCA_978015185	86.58	3.538	8.32	40.3	4.79	5.4	734	3,790	41
MAG_As_31	Bacteria; Pseudomonadota; Gammaproteobacteria; Burkholderiales; Methylophilaceae; Methylotenera	GCA_978015135	97.3	1.652	25.98	47.8	2.22	10	201	2,094	31
MAG_As_32	Bacteria; Actinomycetota; Actinomycetota; UBA4738; CALAEC01	GCA_978015355	91.45	0.598	14.10	71.2	1.97	39	77	2,004	50

These MAGs provide access to genomes of As-impacted paddy soil microbiomes, providing insights into the metabolic potential of impacted organisms in nutrient cycling and metal transport under As stress.

## Data Availability

Raw sequence reads are available in NCBI SRA accessions: SRR19581190 (S4), SRR19581315 (S8), SRR36204389 (A0), SRR36204388 (A6), and SRR36204387 (A9) (BioProject: PRJNA1367948/PRJNA1424688 and PRJNA686650). Draft genomes are available under ENA Project: PRJEB106139.
